# Dietary ω-6 polyunsaturated fatty acid arachidonic acid increases inflammation, but inhibits ECM protein expression in COPD

**DOI:** 10.1186/s12931-018-0919-4

**Published:** 2018-11-03

**Authors:** Sandra Rutting, Michael Papanicolaou, Dia Xenaki, Lisa G. Wood, Alexander M. Mullin, Philip M. Hansbro, Brian G. Oliver

**Affiliations:** 1 Respiratory Cellular and Molecular Biology, Woolcock Institute of Medical Research, The University of Sydney, Sydney, Australia; 2 Priority Research Centre for Healthy Lungs, Hunter Medical Research Institute and The University of Newcastle, Newcastle, NSW Australia; 3 School of Life Sciences, University of Technology Sydney, Sydney, Australia

**Keywords:** COPD, ω-6 PUFAs, Airway inflammation, Remodelling, Human pulmonary fibroblasts

## Abstract

**Background:**

The obesity paradox in COPD describes protective effects of obesity on lung pathology and inflammation. However, the underlying relationships between obesity, diet and disease outcomes in COPD are not fully understood. In this study we measured the response to dietary fatty acids upon markers of inflammation and remodelling in human lung cells from people with and without COPD*.*

**Methods:**

Pulmonary fibroblasts were challenged with ω-3 polyunsaturated fatty acids (PUFAs), ω-6 PUFAs, saturated fatty acids (SFAs) or the obesity-associated cytokine TNFα. After 48–72 h release of the pro-inflammatory cytokines interleukin (IL)-6 and CXCL8 was measured using ELISA and mRNA expression and deposition of the extracellular matrix (ECM) proteins fibronectin, type I collagen, tenascin and perlecan were measured using qPCR or ECM ELISA, respectively.

**Results:**

Challenge with the ω-6 PUFA arachidonic acid (AA), but not ω-3 PUFAs or SFAs, resulted in increased IL-6 and CXCL8 release from fibroblasts, however IL-6 and CXCL8 release was reduced in COPD (*n = 19*) compared to non-COPD (*n = 36*). AA-induced cytokine release was partially mediated by downstream mediators of cyclooxygenase (COX)-2 in both COPD and non-COPD. In comparison, TNFα-induced IL-6 and CXCL8 release was similar in COPD and non-COPD, indicating a specific interaction of AA in COPD. In patients with or without COPD, regression analysis revealed no relationship between BMI and cytokine release. In addition, AA, but not SFAs or ω-3 PUFAs reduced the basal deposition of fibronectin, type I collagen, tenascin and perlecan into the ECM in COPD fibroblasts. In non-COPD fibroblasts, AA-challenge decreased basal deposition of type I collagen and perlecan, but not fibronectin and tenascin.

**Conclusions:**

This study shows that AA has disease-specific effects on inflammation and ECM protein deposition. The impaired response to AA in COPD might in part explain why obesity appears to have less detrimental effects in COPD, compared to other lung diseases.

**Electronic supplementary material:**

The online version of this article (10.1186/s12931-018-0919-4) contains supplementary material, which is available to authorized users.

## Introduction

More than two billion people around the world are overweight or obese, classified by a body mass index (BMI) greater than, or equal to, 25 or 30 kg/m^2^, respectively [1]. This global obesity epidemic is associated with many chronic diseases and recently, its role in lung disease, including chronic obstructive pulmonary disease (COPD), has received new interest. Obesity is becoming more common in mild-to moderate COPD with a prevalence that is generally higher than the general population [2–4]. In the general population, obesity has a major negative impact on health outcomes. However paradoxically, mild-to moderate obesity in moderate to severe COPD is reported to have protective effects on survival, lung function decline and exacerbations [5–8]. The underlying mechanisms of the protective effects of obesity in COPD are currently poorly understood.

In COPD, structural changes to the airways, also known as airway remodelling, occur. A characteristic feature of airway remodelling is airway wall thickening, which is due to an increase in connective tissue. Increased airway wall thickness is associated with frequent exacerbations and symptoms of chronic bronchitis [9, 10]. Connective tissue is composed of scaffolding proteins termed the extracellular matrix (ECM). Perlecan, fibronectin, collagens and tenascin are ECM proteins and their presence is associated with remodelling and/or inflammation in the airways. Tenascin and fibronectin have been shown to be increased in COPD [11]. Interestingly, obesity has also been shown to affect airway remodelling, with BMI being negatively associated with emphysema and positively associated with airway wall thickness [12]. This finding corresponds with the ‘*blue bloater’* phenotype of obese COPD patients, who are more likely to have more bronchitis and less emphysema [9, 13]. Although the exact mechanisms that drive remodelling are still undefined, ongoing chronic inflammatory processes are likely to contribute.

In COPD, airway inflammation is characterized by increased numbers of neutrophils, macrophages, and CD8 + −T lymphocytes, as well as increased levels of interleukin (IL)-6 and CXCL8 in the airways [14, 15]. Neutrophils and CXCL8 levels, in particular, are associated with COPD exacerbations [15–17]. Neutrophils are also strongly implicated in causing chronic bronchitis and the destruction of lung tissue in emphysema, through the production of reactive oxygen metabolites and tissue damaging enzymes [16]. Obesity itself is associated with chronic systemic low-grade inflammation, with increased levels of serum IL-6 and TNFα, produced by adipose tissue [18, 19]. Epidemiological evidence suggests a role for diet in the prevention and management of COPD. Increased intake of certain nutrients, such as vitamin E, D and C and ω-3 polyunsaturated fatty acids (PUFAs) are positively associated with lung function in the general population [20, 21]. In addition, epidemiologic studies have demonstrated that increased intake of these nutrients is associated with a decreased risk of COPD development [20]. These effects are thought to be the result of anti-oxidant and anti-inflammatory properties of these nutrients. Little is known about effects of the Western diet in COPD. The Western diet contributes to obesity, being high in energy from macronutrients, including saturated fatty acids (SFAs) and ω-6 PUFAs. These fatty acids are shown to affect inflammatory processes and have predominantly been associated with pro-inflammatory effects and negatively associated with outcomes in other lung diseases such as asthma [22, 23]. However, the effects of these fatty acids in COPD have not been investigated. ω-3 PUFAs and SFAs affect inflammation by modifying toll-like receptor 4 (TLR4) signalling, whereas ω-6 PUFAs affect inflammation through TLR4-indepenent (independent) mechanisms [24].

A clear causal relation between obesity, diet and disease outcomes in COPD is yet to be proven, but the available data suggest a link between these factors and it is important to understand their effects on airway inflammation and remodelling in COPD. Pulmonary fibroblasts are the major structural cell of the airway and play a crucial role in tissue homeostasis, the production of pro-inflammatory cytokines and ECM proteins and, therefore, are likely to contribute to airway inflammation and remodelling [25, 26]. This study investigated whether pulmonary fibroblasts derived from COPD versus non-COPD patients differ in their inflammatory response to dietary fatty acids (ω-6 PUFAs, ω-3 PUFAs and SFAs) and the obesity-associated cytokine TNFα in vitro*.* Also, the effect of BMI on this response was assessed. Secondly, this study investigated whether dietary fatty acids affect the expression and deposition of ECM proteins in fibroblasts.

## Methods and materials

### Subjects

Primary fibroblasts were isolated from the parenchyma of lungs from patients undergoing lung transplantation or lung resection for thoracic malignancies from a total of *n = 32* donors with COPD, and a total of *n = 50* donors with lung disease other than COPD. The diagnosis of disease was made by thoracic physicians according to current guidelines. Approval for all experiments with human lung was provided by the Human Ethics Committees of the University of Sydney and the Sydney South West Area Health Service. Table 1 shows a summary of the patient demographics.

**Table 1:** Summary of patient demographics

All patients *n* = 82			
	Non-COPD (*n* = 50)		COPD (*n* = 32)
Characteristics	Resection for thoracic malignancy (*n* = 17)	End stage lung disease other than COPD (*n* = 33)	
Pathology	–	1. IPF (*n* = 17) (51.5%) 2. Pulmonary hypertension (*n* = 4) (12.1%) 3. Eisenmenger’s syndrome (*n* = 2) (6.1%) 4. BOS (*n* = 2) (6.1%) 5. Bronchiectasis *(n* = 2) (6.1%) 6. Other (*n* = 6) (18.2%)	1. Emphysema or COPD (*n* = 28) (87.5%) 2. α1-antitrypsin deficiency (*n* = 4) (12.5%)
Sex (n) Female/Male	13/4	11/22	13/19
Mean age (years) (SD)	61.0 (9.7)	55.5 (13.4)	59.1 (8.7)
Mean BMI (kg/m^2^)(SD)	25.0 (5.2)	25.4 (5.3)	23.8 (4.0)
Smokers/non-smokers/*unk* *(% smokers)*	12/2/3 (70.5%)	15/9/9 (45%)	28/2/2 (87.5%)

*COPD* Chronic obstructive pulmonary disease, *IPF* Idiopathic pulmonary fibrosis, *BOS* Bronchiolitis obliterans syndrome, *unk* data Unknown, *SD* Standard deviation, *BMI* Body mass index

### Cell culture

Isolation of pulmonary fibroblasts was performed, as previously described by Krimmer et al. (2013) [27]. Cells were seeded in 12-well plates at a density of 6.2 × 10^4^ cells/mL in DMEM containing 5% fetal bovine serum (FBS) and 1% antibiotic-antimycotic (Gibco, Grand Island, New York, US). When the cells reached 80% confluency, they were serum starved by incubation in DMEM (Gibco, Grand Island, New York, US) supplemented with 0.1% bovine serum albumin (BSA) (Sigma Aldrich, Castle Hill, NSW, Australia) and 1% antibiotic-antimycotic for 24 h prior to stimulation. All experiments were carried out using fibroblasts between passage 2 and 6.

### Preparation of BSA-conjugated fatty acids

Stock solutions of 0.5 M ω-3 PUFA (docosahexaenoic acid (DHA)) and SFA (palmitic acid (PA)) and 0.3 M ω-6 PUFA (arachidonic acid (AA)) (Sigma Aldrich) were prepared in 100% EtOH and stored at-20 °C. Working water-soluble solutions of 10 mM were generated by incubating the fatty acids in 10% endotoxin and fatty acid-free BSA (Sigma Aldrich), as previously described by Gupta et al. (2012) and Pillon et al. (2012) [28, 29]. These solutions were further diluted in cell culture medium to obtain final concentrations of 10 and 100 μM. These concentrations are based on physiological concentrations and other in vitro studies [30–33].

### Treatment of cells with dietary fatty acids and TNFα

Pulmonary fibroblasts from COPD and non-COPD patients were stimulated with 10 and/or 100 μM AA, DHA, PA, or TNFα (1 ng/ml) or vehicle (EtOH/BSA/cell culture medium) and compared to untreated controls. All cells were incubated at 37 °C with 5% CO_2_ for 6, 9, 24, 48 or 72 h. Total RNA or cell-free supernatants were collected and stored at − 20 °C until further analysis.

### Treatment of cells with indomethacin, celecoxib and dexamethasone

Pulmonary fibroblasts from COPD and non-COPD patients were treated with the non-selective cyclooxygenase (COX)-inhibitor, indomethacin (1 μM), the COX-2 selective inhibitor, celecoxib (0.01-1 μM) or corticosteroid, dexamethasone (0.1-1 μM) (all Sigma-Aldrich) for 60 min prior to challenge with dietary fatty acids.

### Determination of IL-6, CXCL8 and PGE2 levels

Levels of IL-6 and CXCL8 in cell culture supernatants were measured using sandwich ELISA. The amount of IL-6 release was assessed with optimized anti-IL-6 antibody pairs from BD pharmingen, BD, Franklin Lakes, NJ. A specific kit for CXCL8 was purchased from R&D Systems (Minneapolis, Minnesota, USA) and used according to the manufacturer’s instructions. PGE2 levels were measured by enzyme immunoassay according to the manufacturer’s instructions (R&D systems).

### Cytotoxicity assay

Cell toxicity was estimated using a lactate dehydrogenase (LDH) assay according to the manufacturer’s instructions (Sigma-Aldrich).

### Determination of COX-2, fibronectin, type I collagen and tenascin mRNA expression

COX-2, fibronectin, type I collagen or tenascin mRNA expression in treated and untreated cell cultures was measured by quantitative PCR (qPCR). Total RNA was purified using the ISOLATE II RNA Mini Kit and transcribed into cDNA using the SensiFAST™ cDNA Synthesis Kit (Bioline, Alexandria, Australia). Both kits were used as per the manufacturer’s instructions. qPCR was performed using the StepOne Plus detection system and data were collected and analysed by StepOne software (Applied Biosystems, Melbourne, Australia). Assays were carried out in triplicate using a reaction mixture containing the Bioline SensiFAST Probe Hi-ROX Master Mix, primer for COX-2, fibronectin, type I collagen or tenascin and the ubiquitously expressed ribosomal RNA (18S rRNA) was used as a housekeeping gene. Relative expression and quantification was performed using the 2ΔΔCT method.

### Measurement of ECM protein deposition

Fibronectin, type I collagen, tenascin and perlecan deposition into the ECM was measured by ECM ELISA using optimised monoclonal mouse-anti human perlecan (2 μg/ml) (ThermoFisher Scientific), type I collagen (2 μg/ml)(Sigma-Aldrich), fibronectin (0.5 μg/ml) (Merck, Bayswater, Australia) and tenascin (0.5 μg/ml) (Sigma-Aldrich) antibodies as previously described by Kuo et al. (2011) [34].

### Statistical analysis

Statistical analysis was conducted using GraphPad Prism version 7 software (GraphPad Software, San Diego, CA). After testing for normal distribution and equal variance, comparisons of the data were carried out by a paired t-test or Analysis of variance (ANOVA) with repeated measures followed by a Bonferroni post-test, where appropriate. All data on bar graphs are presented as mean ± standard error of the mean (SEM), unless otherwise specified. A probability (*p*) value of less than or equal to 0.05 was considered significant.

## Results

### Reduced arachidonic acid-induced cytokine release in COPD versus non-COPD

**Fig. 1:**
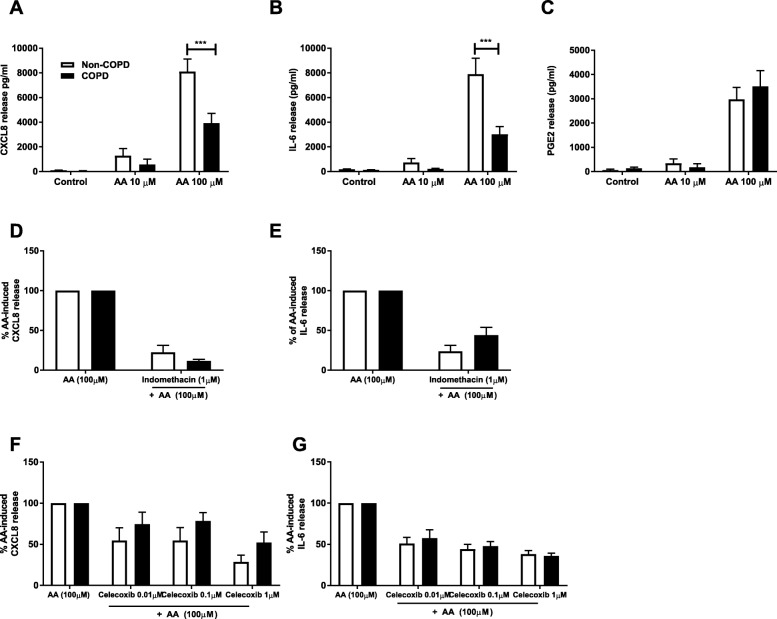
Reduced cytokine release upon arachidonic acid challenge in COPD versus non-COPD. Pulmonary fibroblasts from COPD (*n = 11–19)* and non-COPD patients (*n = 12–36*) were unstimulated (control) or challenged with ω-6 PUFA arachidonic acid (AA) in 0.1% BSA-DMEM (10 and 100 μM) for 48 h. Cell free supernatants were collected and CXCL8 (**a**), IL-6 (**b**) and PGE2 (**c**) release was measured using ELISA or enzyme immunoassay. Other cells from COPD (*n* = 6–7) and non-COPD (*n* = 6–7) patients were pre-treated with indomethacin (1 μM) (**d, e**) or celecoxib (0.01-1 μM) (**f, g**) for 60 min prior to challenge with AA (100 μM) in 0.1% BSA-DMEM for 48 h. Cell free supernatants were collected and CXCL8 (**d, f**) and IL-6 (**e, g**) release was measured using ELISA. Data are expressed as mean ± standard error of the mean (**a-c**) or as % of AA-induced cytokine release ± standard error of the mean (**d-g**). Two-way ANOVA with Bonferroni post-hoc testing was used to determine statistical significance. Significance is represented as *** (*p* < 0.001)

To assess the inflammatory response to dietary fatty acids in pulmonary fibroblasts from COPD versus non-COPD patients, cells were treated with 10 and 100 μM of AA, DHA or PA for 48 h and CXCL8 and IL-6 release was measured. Challenge with DHA or PA did not induce cytokine release from fibroblasts from both COPD and non-COPD patients (*data not shown*). However, challenge with AA resulted in increased CXCL8 and IL-6 release in both groups. Interestingly, CXCL8 and IL-6 release was greater in the non-COPD group (*n* = 36) compared to the COPD group (*n* = 19) (*p* < 0.001) (Fig. 1a and b). The non-COPD group consists of patients with non-smoking related end-stage lung diseases such as pulmonary hypertension and idiopathic pulmonary fibrosis, as well as cells from macroscopically normal lung tissue obtained from resection surgery. As it was possible that pulmonary fibroblasts from patients with disease such as pulmonary hypertension and idiopathic pulmonary fibrosis had a differential response to fibroblasts from macroscopically normal lung tissue we investigated if they respond differently to AA. Fibroblasts derived from patients with non-smoking related end-stage lung disease (*n = 24*) and patients who were going lung resection for thoracic malignancies (*n = 12*) had similar AA-induced cytokine release (Additional file 1: Fig. S1). These results justify combining these two groups in the non-COPD group and indicate a disease-specific effect of COPD, rather than an effect of smoking.

### The response to arachidonic is partially mediated through cyclooxygenase with no differences between COPD and non-COPD

AA can act as a bio-active molecule, and is the precursor that is metabolized by COX to produce prostaglandins, including PGE2. Prostaglandins are known to play a key role in the generation of inflammatory responses. Since the response to AA is different in COPD versus non-COPD, we next assessed whether AA-induced cytokine release is prostaglandin-dependent and whether there are differences in the effect of inhibition of COX on AA-induced cytokine release. We measured the release of PGE2 and found that PGE2 levels are increased upon challenge with AA 100 μM in both COPD (*n* = 11) and non-COPD (*n* = 12) fibroblasts, with no differences between the two groups (Fig. 1c). The non-selective COX-inhibitor indomethacin and the COX-2 selective inhibitor, celecoxib at a concentration of 1 μM both partially suppressed AA-induced IL-6 and CXCL8 release. There were no differences in the percentage of inhibition of AA-induced cytokine release in the COPD versus the non-COPD group at all concentrations of indomethacin or celecoxib used (Fig. 1d, e, f and g). These results show that the response to AA in fibroblasts is partially mediated through downstream mediators of COX-2, and partially mediated through COX-2 independent mechanisms.

### Simular AA-induced COX-2 mRNA expression in COPD and non-COPD

**Fig. 2:**
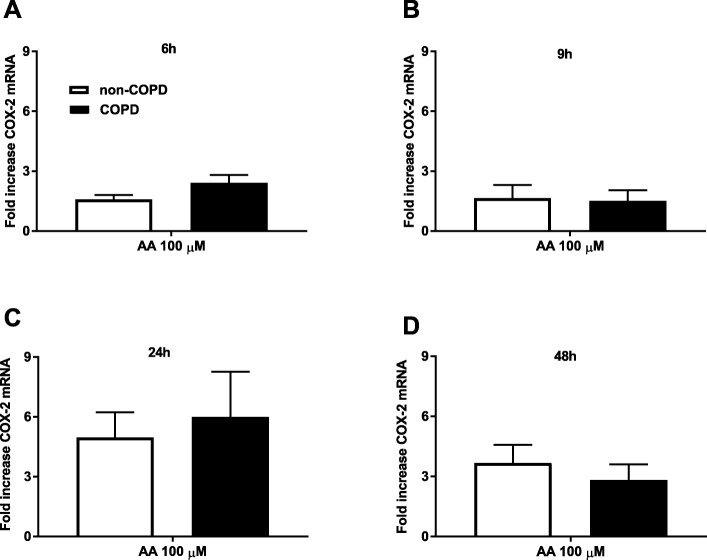
Similar arachidonic acid-induced COX-2 mRNA expression in COPD and non-COPD. Pulmonary fibroblasts from COPD (*n = 5)* and non-COPD patients (*n = 5*) were unstimulated (control) or challenged with ω-6 PUFA arachidonic acid (AA) in 0.1% BSA-DMEM (100 μM) for 6 (**a**), 9 (**b**), 24 (**c**) or 48 (**d**) hours. Total RNA was extracted and cyclooxygenase (COX)-2 mRNA expression was measured using qPCR. The results are normalized to the endogenous control (18S rRNA), and presented as fold change from control (*t* = 0 h) ± standard error of the mean. Unpaired t-test was used to determined statistical significance. There were no statistical differences

To assess whether differences in COX-2 expression could explain the differential response to AA in COPD and non-COPD fibroblasts, we measured COX-2 mRNA expression upon challenge with AA. An AA-induced increase in COX-2 mRNA expression was observed after 6 h with COX-2 mRNA expression being maximal at 24 h. There were no differences in COX2 mRNA expression between COPD (*n* = 5) and non-COPD (*n* = 5) fibroblasts at any time-point (Fig. 2), thereby further confirming that differences in COX-2 expression is not the mechanism by which the differential response to AA occurs.

### Arachidonic acid does not cause cytotoxicity in COPD and non-COPD fibroblasts

**Fig. 3:**
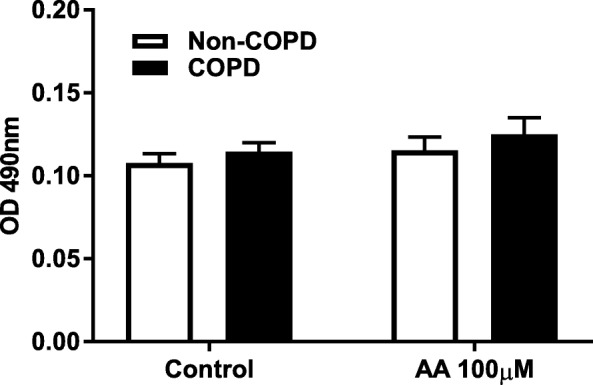
Arachidonic acid does not cause cytotoxicity in COPD and non-COPD fibroblasts. Pulmonary fibroblasts from COPD (*n = 7)* and non-COPD patients (*n = 9*) were unstimulated (control) or challenged with ω-6 PUFA arachidonic acid (AA) in 0.1% BSA-DMEM (100 μM) for 48 h. Cell free supernatants were collected and cell viability was estimated using lactate dehydrogenase (LDH) activity assay. Data is expressed as the absorbance (OD) at 490 nm ± standard error of the mean. Two-way ANOVA with Bonferroni post-hoc testing was used to determine statistical significance. There were no statistical differences

To investigate whether reduced viability is the cause of the impaired response of COPD fibroblasts to AA, we performed a lactate dehydrogenase (LDH) assay and found that AA does not cause cytotoxicity in both COPD (*n* = 7) and non-COPD (*n* = 9) fibroblasts (Fig. 3).

### No differential response to TNFα in COPD versus non-COPD

**Fig. 4:**
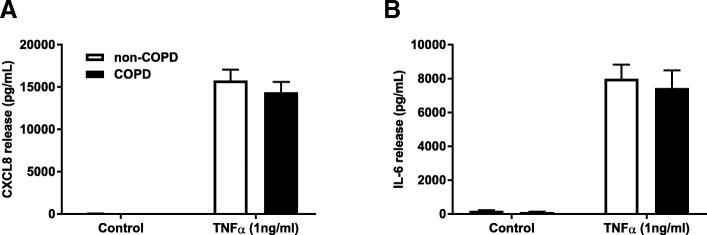
Similar cytokine release upon TNFα challenge in COPD versus non-COPD. Pulmonary fibroblasts from COPD (*n = 15*) and non-COPD patients (*n = 27*) were unstimulated (control) or challenged with TNFα in 0.1% BSA-DMEM (1 ng/ml) for 48 h. Cell free supernatants were collected and CXCL8 (**a**) and IL-6 (**b**) release was measured using ELISA. All data are represented as mean ± standard error of the mean. Two-way ANOVA with Bonferroni post-hoc testing was used to determine statistical significance. There were no statistical differences

We also assessed the inflammatory response to TNFα, which is a pro-inflammatory cytokine and known to be elevated in obese individuals. TNFα induced IL-6 and CXCL8 release from fibroblasts from COPD (*n = 15*) and non-COPD patients (*n = 27*), but there was no difference between the two groups (Fig. 4).

### Dexamethasone suppresses AA-induced cytokine release in COPD and non-COPD

**Fig. 5:**
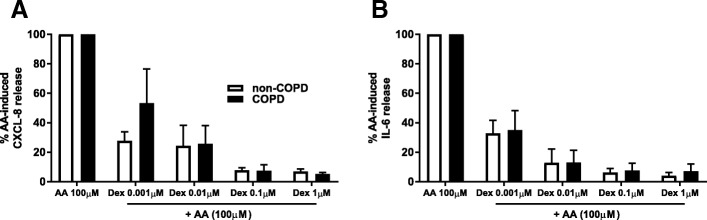
Dexamethasone suppresses AA-induced cytokine release in COPD and non-COPD. Pulmonary fibroblasts from COPD (*n* = 3) and non-COPD (*n* = 6) patients were pre-treated with dexamethasone (0.001 - 1 μM) for 60 min prior to challenge with AA (100 μM) in 0.1% BSA-DMEM for 48 h. Cell free supernatants were collected and CXCL8 (**a**) and IL-6 (**b**) release was measured using ELISA. All data are expressed as % of AA-induced cytokine release ± standard error of the mean. Two-way ANOVA with Bonferroni post-hoc testing was used to determine statistical significance. There were no statistical differences

We next investigated the effects of a steroid, dexamethasone. Steroids have beneficial effects in many inflammatory diseases by exerting their anti-inflammatory effects through inhibiting multiple signal transduction pathways. Dexamethasone (1 μM) almost fully suppressed AA-induced IL-6 and CXCL8 release (Fig. 5) in both groups. Dexamethasone at the lowest concentration of 0.001 μM caused 72% inhibition of AA-induced CXCL8 in the non-COPD cells, whilst only 47% inhibition occurred in the COPD cells, suggesting a reduced steroid sensitivity in COPD versus non-COPD cells.

### Body mass index does not affect the production of inflammatory cytokines

**Fig. 6:**
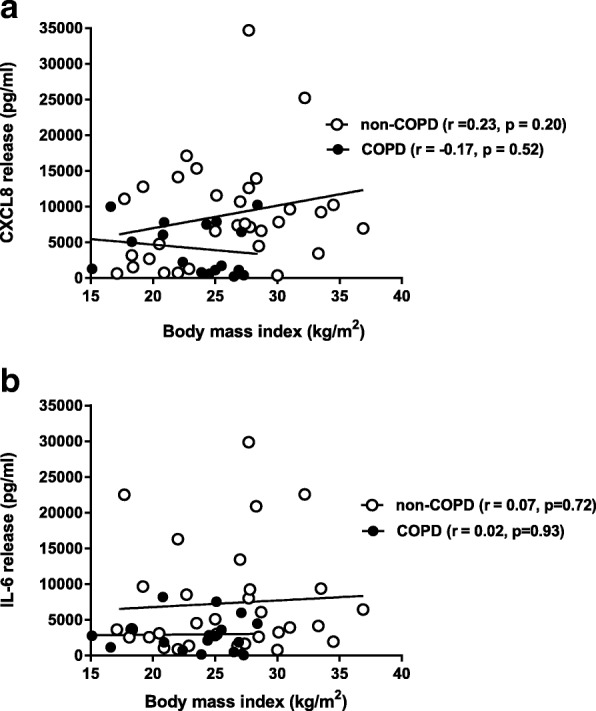
No correlation between BMI and arachidonic acid induced-CXCL8 and IL-6 release. Body mass index (BMI) was correlated with CXCL8 (**a**) and IL-6 (**b**) release upon challenge with arachidonic acid (AA) (100 μM), in fibroblasts from both COPD (*n = 18)* and non-COPD patients (*n = 32)*. The correlation coefficient (r) was determined using linear regression (Pearson analysis). There were no correlations between BMI and CXCL8 or IL-6 release

Since BMI is associated with clinical outcomes in COPD, we investigated if BMI affects the production of inflammatory cytokines upon challenge with AA (100 μM). We found no associations between CXCL8 or IL-6 release and BMI in either the non-COPD (*n = 32*) or the COPD group (*n = 18*) (Fig. 6).

### Arachidonic acid reduces basal ECM protein mRNA expression and deposition

**Fig. 7:**
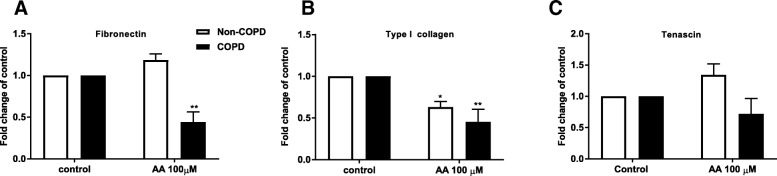
Reduced basal fibronectin and type I collagen expression upon arachidonic acid challenge. Pulmonary fibroblasts from COPD patients (*n = 5*) and non-COPD (*n = 3–4*) were unstimulated (control) or challenged with the ω-6 PUFA arachidonic acid (AA) in 0.1% BSA-DMEM (10 and 100 μM) for 48 h. Total RNA was collected and fibronectin (**a**), Type I collagen (1A2) (**b**) and tenascin (**c**) were detected using real time PCR array. The results are normalized to the endogenous control (18S rRNA), and presented as fold change from control ± standard error of the mean. Challenge with AA is compared to control using a Two-way ANOVA with LSD fisher’s test. Significance is represented as * (*p* < 0.05) and ** (*p* < 0.01)

Since lung pathology is different in obese COPD compared to non-obese COPD, we assessed whether challenge with dietary fatty acids leads to changes in the expression and deposition of ECM proteins. Challenge with AA (100 μM) resulted in significantly reduced fibronectin and type I collagen mRNA expression compared to constitutive levels (*n* = 5, *p* < 0.01) (Fig. 7a and b) in COPD fibroblasts. There was no effect on TNC mRNA expression (Fig. 7c). The effect of AA on fibronectin mRNA expression was specific for COPD cells, whereas AA also decreased basal expression of type I collagen in non-COPD cells *(n* = 5, *p* < 0.05). Challenge with DHA or PA did not affect ECM protein expression (*data not shown*) in COPD and non-COPD cells.

**Fig. 8:**
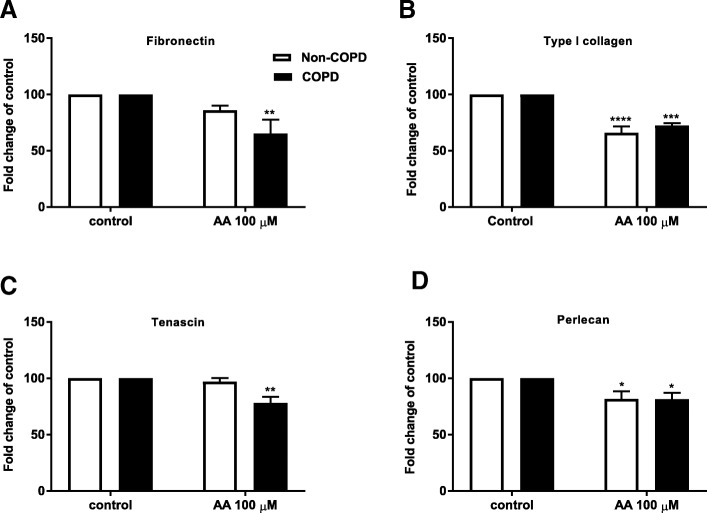
Reduced basal fibronectin, type I collagen, tenascin and perlecan deposition upon arachidonic acid challenge. Pulmonary fibroblasts from COPD (*n = 5–6*) and non-COPD patients (*n = 4–5*) were unstimulated (control) or challenged with the ω-6 PUFA arachidonic acid (AA) in 0.1% BSA-DMEM (100 μM) for 72 h. Deposition of fibronectin (**a**), type I collagen (**b**), tenascin (**c**) and perlecan (**d**) into the extracellular matrix (ECM) was measured by ECM ELISA. All data are expressed at fold change compared to control ± standard error of the mean. Challenge with AA is compared to control using a Two-way ANOVA with LSD fisher’s test. Significance is represented as * (*p* < 0.05), ** (*p* < 0.01), *** (*p* < 0.001) or **** (*p* < 0.0001)

As mRNA levels do not always reflect corresponding protein levels, the deposition of ECM proteins upon challenge with AA 100 μM were also measured using ECM ELISA. Challenge with AA (100 μM) resulted in a reduced basal deposition of all ECM proteins measured (fibronectin, tenascin, type I collagen and perlecan) in COPD fibroblasts (*n* = 5–7) (*p* < 0.05). In non-COPD fibroblasts (*n* = 4–6) challenge with AA reduced the basal deposition of perlecan and type I collagen (*p* < 0.05), but not fibronectin or tenascin (Fig. 8).

## Discussion

This study explored the relationship between dietary fatty acids and airway inflammation and remodelling in COPD using human pulmonary fibroblasts. We found that the ω-6 PUFA AA causes substantial CXCL8 and IL-6 release, and interestingly, this was impaired in fibroblasts from COPD patients. ω-3 PUFAs or SFAs did not induce inflammatory responses in pulmonary fibroblasts from either group.

Pulmonary fibroblasts are the major structural cell of the airway and are found at the interface of the lumen and the blood supply and, therefore, they are directly exposed to components of blood, including nutrients. These cells play a crucial role in tissue homeostasis and the production of pro-inflammatory cytokines and ECM proteins, and, therefore are likely to contribute to airway inflammation and remodelling in COPD [25, 26].

Typically, Western diets are deficient in ω-3 PUFAs and contain excessive amounts of ω-6 PUFAs and SFAs. ω-6 PUFAs and SFAs have predominantly been associated with pro-inflammatory effects, whereas the ω-3 PUFAs are predominantly anti-inflammatory [35]. Western dietary patterns have been associated with an increased risk of newly diagnosed COPD [36, 37] and with greater lung function decline in COPD [38]. We found a pro-inflammatory effect of AA, with impaired cytokine release occurring in COPD cells.

Potential beneficial effects of ω-3 PUFAs have been observed in many diseases, and there is some evidence of a potential role in COPD [39, 40]. Several studies have shown that ω-3 PUFAs reduce inflammation by inhibiting TLR4 signalling [24]. In addition, observational studies have shown that diets rich in fruit, vegetables and ω-3 PUFAs are positively associated with FEV1 and FVC in the general population [41], and reduce the risk of developing of COPD [36, 37, 42, 43]. We found no pro-inflammatory effects of ω-3 PUFAs which is consistent with most literature, however this study did not investigate potential anti-inflammatory effects. Furthermore, SFAs did not induce cytokine release from pulmonary fibroblasts. SFAs, including PA, initiate innate immune responses via TLR 2 and 4 signalling [44–48]. The non-responsiveness to SFAs in this study may be explained by the lack of functional TLR4/CD14 signalling in pulmonary fibroblasts [49, 50]*.*

The majority of the patients in the COPD and non-COPD group were smokers, however the percentage of smokers was higher in the COPD group. The non-COPD group consists of patients with non-smoking related end-stage lung diseases, as well as cells from macroscopically normal lung tissue obtained from resection surgery. We confirmed that there was no difference in the response to AA between these two groups. Furthermore, TNFα induced similar cytokine release in the COPD and non-COPD group. Taken together this shows that COPD fibroblasts have a specific impairment in their response to AA, independent of smoking history.

The current study measured IL-6 and CXCL8 as these mediators are important in the pathogenesis of COPD and are increased in serum and BAL fluid of COPD patients [15, 17]. IL-6 is a marker of systemic inflammation, a predictor of mortality and is negatively correlated with lung function [15, 51]. CXCL8 is a potent neutrophil chemoattractant and activates neutrophils, leading to the secretion of reactive oxygen metabolites, inflammatory cytokines and tissue damaging enzymes. In this study AA induced lower levels of IL-6 and CXCL-8 in COPD versus non-COPD pulmonary fibroblasts, suggesting that in COPD meals rich in ω-6 PUFAs are not as potent in the induction of inflammatory responses compared to other chronic lung diseases.

AA affects inflammation through TLR4 independent mechanisms. It acts as a bio-active molecule and is converted into eicosanoids, including prostaglandins, through metabolism by COX. Prostaglandins are known to play a key role in the generation of inflammatory responses [52] and have been shown to affect cytokine production in immune and lung cells [53, 54]. AA induced prostaglandin E2 (PGE2) release, but there was no difference between COPD and non-COPD fibroblasts. The non-selective COX-inhibitor, indomethacin, and the COX-2 selective inhibitor, celecoxib both partially suppressed AA-induced IL-6 and CXCL8 release showing that cytokine induction also occurs via COX-2 independent mechanisms. There were no differences in the percentage of inhibition between COPD and non-COPD fibroblasts. In addition, there were no differences in AA-induced COX-2 mRNA expression between COPD and non-COPD cells.

Since BMI is associated with clinical outcomes in COPD, we investigated whether obesity itself affects the response to AA in pulmonary fibroblasts. We found no effect of BMI on AA-induced CXCL8 or IL-6 release from pulmonary fibroblasts, in both groups. Epidemiological studies have reported that moderate obesity in moderate to severe COPD has protective effects on survival, lung function decline and exacerbations [5–8]. Cai et al. (2003) showed beneficial effects of high fat diet versus low fat diet on lung function in COPD. These effects of obesity are contradictory to the known deleterious effects of obesity in the general population and other diseases [55]. McDonald et al. (2016) investigated the effect of exercise and a low-energy diet in obese COPD patients. They found some clinically significant improvements on COPD outcomes including health status, but no effects on inflammatory markers and lung function [56]. Clearly, more studies are needed to understand the effects of obesity and diet in COPD.

We investigated the ability of dietary fatty acids to affect ECM protein mRNA expression and deposition. AA, but not DHA or PA reduced basal fibronectin and type I collagen mRNA expression and fibronectin, type I collagen, tenascin and perlecan deposition in COPD fibroblasts. The inhibitory effect of AA on ECM protein expression and deposition was less substantial in non-COPD cells.

Fibronectin and tenascin are increased in COPD airways and their presence is correlated with airway remodelling and/or inflammation and is negatively correlated with FEV1 [11, 57, 58]. The effect of obesity on airway remodelling is not well established. However, BMI has been negatively associated with the severity of emphysema, independent of gender, age and smoking history and positively associated with airway wall thickness [12]. Our results show that AA suppresses the basal deposition of fibronectin, type I collagen, tenascin and perlecan suggesting that AA and possibly other dietary factors could play a role in the regulation of ECM deposition in COPD. ECM proteins play an essential role in maintaining tissue homeostasis affecting many cellular processes including proliferation, migration and repair [59]. Decreased levels of ECM proteins could lead to inadequate repair mechanisms in COPD.

One limitation of our study is the lack of pulmonary fibroblasts from obese and severely obese COPD and non-COPD patients. In addition, the BMI was lower in the COPD group compared to the non-COPD group. This occurred by chance, as we did not select patients on the basis of BMI; rather we used samples as they were available. It may be that the protective effects of obesity account for the lack of samples from the obese COPD population, as if patients have reduced severity of COPD they would not need lung transplantation. Interestingly, a recent meta-analysis has shown that BMI is also inversely associated with lung cancer [60], which could explain the limited resection samples from obese patients with a thoracic malignancy.

## Conclusion

Our study demonstrates that ω-6 PUFA AA, but not ω-3 PUFA DHA or SFA PA, affects inflammatory processes and ECM deposition in COPD. We found that whilst AA increases inflammation, pulmonary fibroblasts from patients with COPD had a reduced response to AA in comparison to cells from people without COPD. Obesity itself was not associated with the inflammatory response. Moreover, we found that AA had a more substantial inhibitory effect on basal ECM-protein expression and deposition in COPD cells compared to non-COPD cells. This study suggest that the dietary fatty acid AA and possibly other dietary components have disease-specific effects and could explain differential effects of high fat diets in different lung diseases. The impaired response to AA in COPD might in part explain why obesity appears to have less detrimental effects in COPD, compared to other lung diseases.

## Additional file

Additional file 1: **Figure S1.** Similar response to arachidonic acid in patients with non-smoking related end-stage lung disease and patients who underwent lung resection for thoracic malignancies. (DOC 153 kb)

## Abbreviations

18sRNA: 18S ribosomal RNA
AA: Arachidonic acid
ANOVA: Analysis of variance
BMI: Body mass index
BSA: Bovine serum albumin
COPD: Chronic obstructive pulmonary disease
COX: Cyclooxygenase
CXCL8: Chemokine (C-X-C motif) ligand 8
DHA: Docosahexaenoic acid
ECM: Extracellular matrix
FBS: Fetal bovine serum
IL-6: Interleukin 6
PA: Palmitic acid
PUFA: Polyunsaturated fatty acid
qPCR: Quantitative polymerase chain reaction
SEM: Standard error of the mean
SFA: Saturated fatty acid
