# Supplementary material for: Dietary ω-6 polyunsaturated fatty acid arachidonic acid increases inflammation, but inhibits ECM protein expression in COPD
Source: Respir Res. 2018 Nov 3;19:211. doi: 10.1186/s12931-018-0919-4 (PMC6215599; doi:10.1186/s12931-018-0919-4)
Supplement: Supplementary file 1 — Figure S1. Similar response to arachidonic acid in patients with non-smoking related end-stage lung disease and patients who underwent lung resection for thoracic malignancies. (DOC 153 kb) [file 12931_2018_919_MOESM1_ESM.doc]

**TITLE:** Dietary ω-6 polyunsaturated fatty acid arachidonic acid increases inflammation, but inhibits ECM protein expression in COPD. Sandra Rutting^1,2^, Michael Papanicolaou^1,3^, Dia Xenaki^1^, Lisa G. Wood^2^, Alexander M. Mullin^1^, Philip M. Hansbro^2^, Brian G. Oliver^1,3^ ^1^ Respiratory Cellular and Molecular Biology, Woolcock Institute of Medical Research, The University
 of Sydney, Sydney, Australia; ^2^ Priority Research Centre for Healthy Lungs, Hunter Medical Research Institute and The University of
 Newcastle, Newcastle, NSW, Australia;
^3^ School of Life Sciences, University of Technology Sydney, Sydney, Australia

**Appendix S1. Supplementary figure 1**

**Figure S1.** **Similar response to arachidonic acid in patients with non-smoking related end-stage lung disease and patients who underwent lung resection for thoracic malignancies.**

**
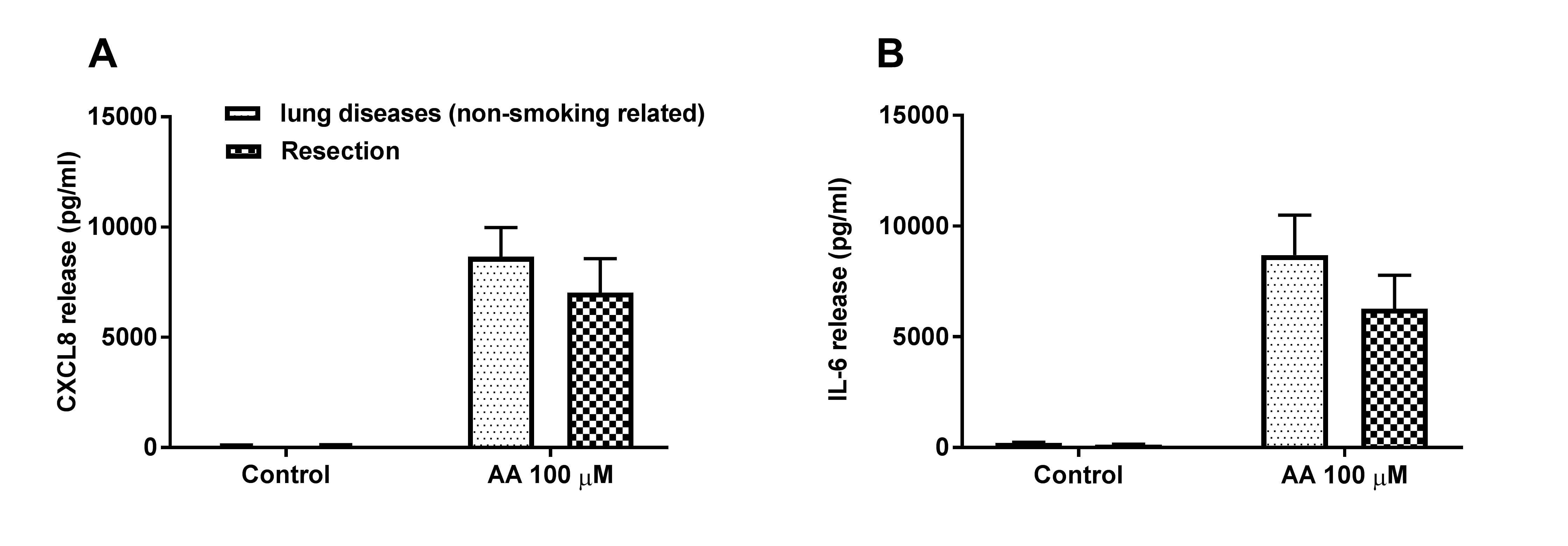
**

Human primary lung fibroblasts from patients with end-stage lung diseases other than COPD (non-smoking related) (*n = 24*) and patients who were undergoing lung resection for thoracic malignancies (*n = 12*) were unstimulated (control) or challenged with ω-6 PUFA arachidonic acid (AA) in 0.1% BSA-DMEM (10 and 100µM) for 48h. Cell free supernatants were collected and CXCL8 **(A)** and IL-6 **(B)** release was measured using ELISA. All data are represented as mean ± standard error of the mean. Two-way ANOVA with Bonferroni post-hoc testing was used to determine statistical significance. There were no statistical differences.
